# Continuous Erector Spinae Plane Analgesia in Kidney Transplant Recipients: A Quality Improvement Project

**DOI:** 10.7759/cureus.39151

**Published:** 2023-05-17

**Authors:** Padmini Vishwanath, Alka Deo, Parimala Balakundi

**Affiliations:** 1 Anaesthesiology, Nephrology-Urology (NU) Hospitals, Bangalore, IND

**Keywords:** perioperative analgesia, regional anaesthesiology, continuous catheter analgesia, chronic kidney failure pain management, erector spinae plane (esp) blockade, transplant, opioid-free analgesia, acute pain

## Abstract

Introduction

Pain management in patients with chronic kidney disease is challenging. Due to impaired kidney function, analgesic options are limited. Postoperative analgesia in transplant recipients is further complicated by their vulnerability to infections, titrated fluid management and optimal haemodynamics to maintain graft function. Erector spinae plane (ESP) blocks have been used successfully in a variety of surgeries. This study is a quality improvement project aiming to assess the efficacy of continuous erector spinae plane catheter analgesia in the postoperative management of kidney transplant recipients.

Methods

We conducted an initial audit over a period of three months. All patients who underwent kidney transplantation under general anaesthesia with erector spinae plane catheters were included. Erector spinae plane catheters were secured prior to induction, and continuous local anaesthetic infusion was maintained postoperatively. Pain scores using the numerical rating scale (NRS) were recorded at intervals in the first 24 hours postoperatively, and supplementary analgesics given were noted. Following satisfactory results from the initial audit, we implemented erector spinae plane catheters as part of multimodal analgesia in transplant patients in our centre. We re-audited all transplants done over the next year to reassess the quality of postoperative analgesia.

Results

Five patients were audited during the initial audit. The average NRS score ranged from 0 at rest to a maximum of 5 during mobilisation. All patients were given only paracetamol to supplement analgesia, and none required opioids. During the re-audit, data was collected on postoperative pain management in 13 subsequent transplants conducted over the next year. The NRS scores ranged from 0 at rest to 6 on mobilisation. Two patients required boluses of fentanyl 25 mcg via the catheter, and the rest reported satisfactory analgesia with paracetamol as needed.

Conclusion

This quality improvement project changed our centre’s practice in managing postoperative pain in kidney transplantations. We switched from securing epidural catheters to erector spinae plane catheters due to better safety profile, minimal use of opioids and lesser adverse effects. We shall continue to re-audit our practices for the best outcomes.

## Introduction

Erector spinae plane (ESP) block is evolving as a popular technique for perioperative analgesia in recent years. Initial reports of its successful use in thoracic surgeries and neuropathic pain led to its use in various abdominal procedures including hepatectomy, nephrectomy and percutaneous nephrolithotomy [1]. As per current literature, ESP block, being an easy technique to perform and having a good safety profile, is a promising technique to be explored, especially in patients with multiple comorbidities. The authors presented the initial audit of this study as a case series on the use of continuous ESP analgesia for postoperative pain in kidney recipients. It was presented as a poster at the virtual Euroanaesthesia Conference 2021.

## Materials and methods

Five patients with end-stage kidney disease who underwent live-related donor kidney transplantation in our centre, between May and September 2021, were included in the initial audit. The re-audit was done in the year that followed between October 2021 and September 2022, and thirteen patients were included. The patients were explained in detail about the technique of the insertion of an ESP catheter, the benefits and associated complications. A written informed consent was obtained.

The ESP catheters were placed preoperatively in the operating room before the induction of general anaesthesia. Standard patient monitoring as per the American Society of Anesthesiologists, including electrocardiogram (ECG), pulse oximetry and noninvasive blood pressure monitors, was connected. Oxygen was administered via nasal prongs. The patients were positioned prone and sedated with intravenous (IV) midazolam 1 mg and IV fentanyl 1 mcg/kg. After sterile skin preparation, a curvilinear ultrasound probe was placed in the right parasagittal plane; transverse processes at the levels of T8-T9 were identified. The erector spinae muscle superficial to it was identified. An 18G Tuohy needle was introduced in the plane with the probe, in the cephalo-caudal direction towards the transverse process. After contacting the transverse process, 20 ml of 0.375% ropivacaine with dexmedetomidine 50 mcg was injected. The correct plane was confirmed by the linear spread of the drug lifting the erector spinae muscle off the transverse processes. A 20G epidural catheter was inserted into the needle and advanced 5 cm into the space. After securing the catheter, the patient was positioned supine, and general anaesthesia was induced. Fentanyl 1 mcg/kg, propofol 1-2 mg/kg and atracurium 0.5 mg/kg were administered. Anaesthesia was maintained with oxygen, nitrous oxide and sevoflurane mixture.

A modified Gibson incision was made. Fentanyl 1 mcg/kg was to be administered if patients developed tachycardia at more than 20% of baseline heart rate. However, none of the patients required additional opioids intraoperatively. At the end of the surgery, as abdominal closure began, IV paracetamol 1 g was administered, and ESP catheter infusion was initiated with 0.125% bupivacaine at 12 ml/hour. The patients were then extubated immediately after surgery and transferred to the high-dependency unit.

**Figure 1:**
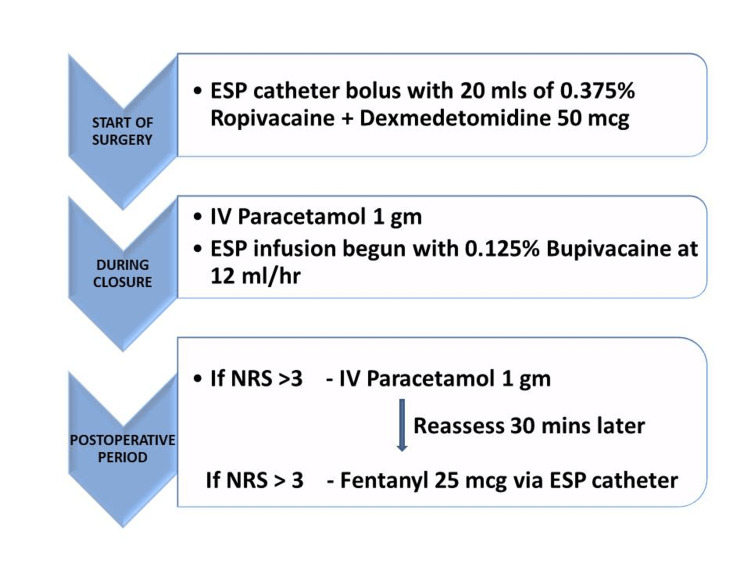
Pain management protocol NRS, numerical rating scale; ESP, erector spinae plane; IV, intravenous

The pain was assessed using the numerical rating scale (NRS). The patient picks a number between 0 and 10, with 0 being ‘no pain’ and 10 being ‘the worst pain imaginable’. NRS scores were recorded at one, four, eight, 12, 18 and 24 hours postoperatively by a trained staff nurse. If patients scored more than 3 on NRS, IV paracetamol 1 g was administered, and the pain was reassessed after 30 minutes. On reassessment 30 minutes later, if the pain persisted (NRS of >3), IV tramadol 50 mg was to be given. During the re-audit, the rescue analgesic was changed to fentanyl 25 mcg given via the catheter with the ongoing local anaesthetic infusion (Figure 1).

## Results

The patient demographics, postoperative NRS scores and use of any supplementary analgesics are shown in Table 1. Four out of the five patients required supplementation with IV paracetamol 1 g once, and only one patient required it twice in the 24 hours following surgery. The NRS scores ranged from 0 during rest to a maximum of 5 during mobilisation. None of the patients required IV tramadol or other opioids. No adverse events were noted.

**Table 1:** Initial audit, trial of ESP catheter: patient demographics, NRS pain scores and supplementary analgesia IV, intravenous; NRS, numerical rating scale; ESP, erector spinae plane

Serial number	Age	Gender	NRS						IV paracetamol (PCT) at hours postop	NRS 30 minutes after PCT
			1 hour	4 hours	8 hours	12 hours	18 hours	24 hours		
1	34	Male	0	1	2	1	2	1	At seven hours	1
2	45	Female	2	2	1	2	1	0	At 14 hours	1
3	47	Male	0	0	2	2	1	1	At 14 hours	2
4	51	Male	2	1	0	3	0	1	6 and 12 hours	1
5	23	Male	0	0	2	1	5	1	18 hours	2

During the re-audit, data from 13 patients was analysed. Rescue analgesic was changed from tramadol to fentanyl 25 mcg to be given via the catheter. The NRS scores ranged from 0 at rest to 6 on mobilisation. Two patients required boluses of fentanyl 25 mcg via the catheter, and the rest reported satisfactory analgesia with paracetamol as needed. The patient demographics, NRS scores and analgesics given are shown in Table 2.

**Table 2:** Re-audit, post implementing ESP catheter in pain management protocol: patient demographics, pain scores and supplementary analgesia IV, intravenous; PCT, paracetamol; NRS, numerical rating scale; ESP, erector spinae plane

Serial number	Age	Gender	NRS						IV PCT 1 g/fentanyl 25 mcg (via catheter)	NRS 30 minutes later
			1 hour	4 hours	8 hours	12 hours	18 hours	24 hours		
1	17	Male	3	4	2	6	3	3	PCT eight hourly + fentanyl 25 mcg at 12 hours	2 and 3
2	30	Female	2	2	2	4	2	2	PCT at 12 hours	2
3	36	Male	2	1	1	1	3	2	PCT at 18 hours	1
4	44	Male	1	1	1	2	1	1	Nil	
5	36	Male	3	2	2	2	3	1	PCT at 18 hours	2
6	39	Female	4	5	3	3	1	2	PCT at four and 12 hours	2 and 2
7	49	Male	2	2	2	2	3	2	PCT at 18 and 24 hours	2 and 1
8	61	Male	3	5	3	2	2	3	PCT at four and 24 hours	2 and 2
9	20	Female	3	3	2	1	1	0	PCT at eight hours	0
10	31	Male	6	2	2	1	1	1	Fentanyl 25 mcg at one hour	2
11	27	Male	4	4	2	1	1	1	PCT given eight hourly	<3
12	27	Male	1	2	2	2	2	2	Nil	
13	58	Male	3	3	5	3	1	1	PCT at eight and 16 hours	3 and 1

## Discussion

Pain management in chronic kidney disease presents many challenges. The altered pharmacology due to the manifestations of chronic kidney disease is due to changes in the volume of the distribution of drugs, altered protein binding, delayed clearance and excretion with a higher risk of the accumulation of metabolites. In a systemic review conducted by Lambourg et al., they reported a high incidence of analgesic consumption in patients with chronic kidney disease and associated adverse effects [2].

Harrison et al. reported a higher incidence of major surgeries and postoperative morbidity in patients with chronic kidney disease [3]. In patients undergoing kidney transplantation, there are further difficulties in perioperative management due to immunosuppression and graft preservation [4].

Nonsteroidal anti-inflammatory drugs (NSAIDs), which are commonly used as part of multimodal analgesia in other patient populations, are contraindicated here. NSAIDS are associated with acute kidney injury and the progression of chronic kidney disease [5]. Epidural analgesia and opioids have been the mainstay in postoperative pain management.

In the background of restricted fluid management, immunosuppression and deranged platelet function, there is a higher risk of complications of epidural analgesia, which include hypotension, epidural abscess or haematomas. The literature on the use of epidurals in kidney transplants is conflicting, with some associating improved graft function [6] and some reporting postoperative acute kidney injury with the use of epidural anaesthesia [7].

The existing literature has emphasised the need to minimise the perioperative use of opioids in kidney transplants [8]. The enhanced recovery after surgery (ERAS) pathway also advocates the use of multimodal analgesia to reduce opioid requirements [9]. Opioids have been associated with delayed recovery, prolonged hospital stay and increased risk of death [2].

Newer regional techniques such as quadratus lumborum and ESP blocks are being explored for effective analgesia, although technical expertise is required [10]. ESP analgesia offers several advantages, which include simple technique, the possibility of performing the procedure in different positions for a myriad of surgeries, lesser sympathectomy-mediated hypotension and fewer reported adverse events. Though the efficacy of visceral analgesia is debated, the presumed mechanism of analgesia by ESP is through the spread of local anaesthetic into the paravertebral and epidural space and around somatic nerves [11,12]. Most of the available literature report the use of ESP in thoracic surgeries and few for abdominal surgeries [1,13]. Minimal data, mainly case reports, is available on its use in kidney transplantation [11,14]. Sharipova et al. conducted a retrospective study comparing the efficacy of ESP catheters with conventional pain management after renal transplants [15]. They concluded that ESP blocks were effective in combination with non-opioid analgesics. In our study, we concur with their findings and successfully demonstrate the use of continuous erector spinae block for effective analgesia postoperatively with the minimal use of opioids.

Our study, being a clinical audit, is limited by a small sample size. Further prospective studies are required to validate the efficacy of erector spinae catheters in kidney transplants. We also need to re-audit our practices regularly to ensure continued good outcomes and assess other parameters such as time to recovery or discharge and overall patient satisfaction scores.

## Conclusions

Multimodal analgesic regimens with minimal opioids may prove beneficial for early mobilisation, recovery and discharge in kidney transplants. The advancement of newer interfascial plane blocks has reduced the use of neuraxial techniques and opioids. In our study, we report the good efficacy of continuous ESP catheter analgesia.

ESP analgesia, being effective and having a good safety profile, is an appealing modality and warrants further investigation into expanding its utility in renal transplantation and other surgeries. Randomised controlled trials on the effectiveness and adverse events, in comparison with epidural analgesia and other modalities, are required.
